# Improving Hand Hygiene Compliance in a Resource-Limited ICU Using a Low-Cost Multimodal Quality Improvement Intervention

**DOI:** 10.3390/healthcare14030363

**Published:** 2026-01-30

**Authors:** Sadia Qazi, Muhammad Amir Khan, Athar Ud Din, Naimat Saleem, Eshal Atif, Muhammad Atif Mazhar

**Affiliations:** 1 Anatomy Department, College of Medicine, Alfaisal University, Riyadh 11533, Saudi Arabia; 2 Department of Internal Medicine, MTI Mardan Medical Complex, Mardan 23200, Pakistan; dramirkhan95@gmail.com (M.A.K.); atharuddin11298@gmail.com (A.U.D.); 3 Khyber Girls Medical College, Peshawar 25100, Pakistan; naimatkhan2389@gmail.com; 4 College of Medicine, Alfaisal University, Riyadh 11533, Saudi Arabia; eatif@alfaisal.edu

**Keywords:** hand hygiene, quality improvement, intensive care unit, WHO Five Moments, infection prevention, audit and feedback, resource-limited settings, patient safety, episode-based audit, non-equivalent dependent variables

## Abstract

**Highlights:**

**What are the main findings?**

- A low-cost multimodal intervention was associated with increased composite hand hygiene compliance in a resource-limited ICU, from 63.1% to 82.0% over a four-week observation period.
- Sensitivity analysis excluding non-applicable moments demonstrated that pure adherence improved from 54.2% to 82.5%, confirming a genuine behavioral change rather than a measurement artifact.
- The largest gains occurred in awareness-dependent WHO moments targeted by the intervention (before touching the patient: +27.0 pp; after touching the patient’s surroundings: +40.0 pp), with smaller changes in non-targeted moments.
- Baseline pure adherence for high-risk moments involving body fluid exposure was substantially lower (12.2%) than that suggested by the primary compliance metric (80.0%), revealing how “compliant by default” scoring can mask poor performance in frequently non-applicable moments.

**What are the implications of the main findings?**

- Pragmatic, workflow-embedded interventions utilizing education, visual cues, feedback, and leadership engagement can achieve measurable short-term compliance improvements without electronic monitoring or additional resources in resource-limited ICU settings.
- Episode-based audits with fixed denominators provide operationally feasible tools for directional process monitoring but should be complemented with sensitivity analyses reporting pure adherence to distinguish true behavioral changes from measurement artifacts.
- Long-term sustainability beyond the four-week observation period remains unknown; extended follow-up at 6–12 months is required to assess the persistence of improvements and the need for ongoing reinforcement.

**Abstract:**

**Background/Objective:** Hand hygiene is a cornerstone of infection prevention; however, compliance is inconsistent in intensive care units (ICUs), particularly in resource-constrained settings. This study evaluated whether a low-cost, multimodal quality improvement intervention could improve process-level hand hygiene compliance using routine, episode-based audits embedded in the ICU practice. **Methods:** We conducted a single-cycle Plan-Do-Study-Act quality improvement project in a 12-bed mixed medical–surgical ICU in Pakistan (December 2023–January 2024). Hand hygiene performance was assessed using the unit’s routine weekly episode-based audit protocol, aligned with the WHO Five Moments framework. A targeted multimodal intervention comprising education, point-of-care visual reminders, audit feedback, and leadership engagement was implemented between the pre- and post-intervention phases (four weeks each). Non-applicable moments were scored as “compliant by default” according to the institutional protocol. A sensitivity analysis was performed excluding these moments to calculate pure adherence. Compliance proportions were summarized using exact 95% Clopper–Pearson confidence intervals without inferential testing. **Results:** A total of 942 audit episodes (471 per phase) generated 4710 moment-level assessments were generated. Composite hand hygiene compliance increased from 63.1% pre-intervention to 82.0% post-intervention [absolute increase: 18.9 percentage points (pp)]. Sensitivity analysis excluding non-applicable moments demonstrated pure adherence improvement from 54.2% to 82.5% (+28.3 pp), confirming a genuine behavioral change rather than a measurement artifact. Compliance improved across all five WHO moments, with the largest gains in awareness-dependent moments targeted by the intervention: before touching the patient (+27.0 pp) and after touching patient surroundings (+40.0 pp). Week-by-week compliance remained stable within both phases, without immediate post-intervention decay. **Conclusions:** A pragmatic, low-cost multimodal intervention embedded in routine ICU workflows was associated with substantial short-term improvements in hand hygiene compliance over a four-week observation period, particularly for awareness-dependent behaviors. Episode-based audit systems can support directional process monitoring in resource-limited critical care settings without the need for electronic surveillance. However, its long-term sustainability beyond one month and generalizability to other settings remain unknown. Sensitivity analyses are essential when using “compliant by default” scoring to distinguish adherence patterns from measurement artifacts.

## 1. Introduction

Hand hygiene is a cornerstone of patient safety; however, compliance in intensive care units remains persistently suboptimal despite decades of guidelines, audits, and education efforts [1,2]. Healthcare-associated infections impose substantial global morbidity, mortality, and economic burden, with ICUs at a particularly high risk owing to invasive procedures, high patient acuity, and frequent staff–patient interactions [3,4,5]. The WHO Five Moments for Hand Hygiene framework has standardized when hand hygiene should occur and how compliance should be assessed [4,5]. While a substantial proportion of these infections are preventable through adherence to hand hygiene protocols [6], their implementation faces significant barriers in resource-limited settings [7]. Multimodal interventions that combine education, reminders, feedback, and leadership support have been shown to be effective in improving adherence and reducing infection rates [8,9,10,11]. Systematic reviews have confirmed that audit and feedback mechanisms can drive professional practice changes when implemented systematically [12]. However, compliance gains remain heterogeneous across settings, staff groups, and hand hygiene moments and often prove modest or short-lived [11,13]. Measurement challenges are compounded by the Hawthorne effect, where observation itself may artificially elevate compliance during the audit periods [13].

This persistent variability reflects a critical methodological gap [14]. Hand hygiene research employs diverse audit approaches, including direct observation, electronic monitoring, and opportunity-triggered counting, with fundamentally different operational definitions of what constitutes an “observation” and how results are calculated. Moreover, observer bias in compliance reporting can systematically inflate adherence estimates, particularly when auditors are aware of intervention implementation or institutional performance targets [15]. These measurement choices shape both the interpretation of compliance data and the perceived magnitude of improvement [16]. Multiple audit methodologies have been proposed, each with distinct trade-offs between feasibility, accuracy, and resource requirements [17]. While many methods implicitly assume discrete, independent events, ICU care unfolds through overlapping workflows, where pragmatic process monitoring must accommodate clinical realities [18]. Episode-based audits, which assess a standardized set of WHO moments during defined care episodes rather than counting all opportunities, offer operational simplicity and workflow compatibility [19]. Despite their widespread use in quality improvement practice, episode-based approaches have received limited methodological scrutiny, particularly regarding how scoring rules for non-applicable moments influence compliance estimates and how clustering effects affect statistical inference.

This methodological opacity has practical consequences in resource-limited settings, where electronic monitoring and continuous surveillance are often infeasible and evidence-based guidance on feasible audit strategies remains scarce. In this context, quality improvement initiatives must balance methodological rigor with operational feasibility [20]. Moreover, ICU hand hygiene failures often reflect moment recognition and situational awareness deficits rather than technical skill gaps [11], suggesting that interventions targeting education and environmental cues may yield selective improvement in awareness-dependent behaviors (e.g., before touching the patient, after touching patient surroundings) compared to procedurally salient moments (e.g., after body fluid exposure risk) that already trigger higher baseline adherence [5,18]. However, few studies have systematically examined whether observed compliance patterns align with intervention targeting or applied pattern-matching principles from quasi-experimental designs to strengthen confidence that changes reflect genuine behavioral responses rather than secular trends or measurement artifacts.

### 1.1. Study Rationale and Contributions

This study addresses three such evidence gaps. First, it provides transparent methodological documentation of an episode-based audit approach, including explicit handling of non-applicable moments, sensitivity analyses examining the impact of scoring rules, and discussion of clustering effects, details that are rarely reported in the hand hygiene QI literature. Second, it demonstrates the feasibility and short-term behavioral response to a low-cost, multimodal intervention specifically designed for a resource-limited ICU setting in Pakistan, contributing context-specific evidence to an evidence base dominated by high-resource settings. Third, it applies the pattern-matching principle from quasi-experimental design to hand hygiene compliance data, showing how selective improvement in intervention-targeted moments (versus stability in non-targeted moments) strengthens confidence that observed changes reflect genuine behavioral responses rather than general trends or measurement artifacts alone.

### 1.2. Study Objectives and Scope

We conducted a single-cycle PDSA quality improvement project using a structured, episode-based hand hygiene audit embedded within routine ICU practice. The study was designed to evaluate the short-term feasibility and immediate behavioral responses to the intervention, not long-term sustainability or clinical outcomes. Specifically, we asked: (1) How does composite hand hygiene compliance change during the four weeks following the implementation of a low-cost multimodal intervention compared to the four weeks prior? (2) How do compliance patterns vary across the five WHO moments before and after the intervention, and do targeted awareness-dependent moments show selective improvement consistent with the intervention focus? We position this work as a pilot evaluation providing a transparent, replicable framework for initiating hand hygiene improvement in resource-limited ICUs, with explicit acknowledgment that longer-term sustainability assessment and outcome linkage remain essential next steps.

## 2. Methods

### 2.1. Study Design and Setting

We conducted a single-cycle quality improvement project using a pre-post Plan-Do-Study-Act (PDSA) design in the 12-bed mixed medical–surgical ICU at Bacha Khan Medical Complex MTI, Swabi, Pakistan (December 2023–January 2024). This design was selected because it enables pragmatic process evaluation embedded within routine clinical workflows without requiring randomization or concurrent controls, consistent with the SQUIRE 2.0 guidelines (Supplementary Materials) for QI reporting [21]. The ICU operates three shifts daily (morning 07:00–15:00, evening 15:00–23:00, and night 23:00–07:00) and is staffed by approximately 30 nurses and 20 interns or nursing students.

The study assessed process-level hand hygiene performance using the hospital’s existing routine weekly audit protocol, aligned with the WHO Five Moments framework. An episode-based audit approach was employed rather than a traditional opportunity-based counting approach because it provides operational simplicity, workflow compatibility, and a fixed denominator structure suitable for rapid-cycle quality improvement in resource-limited settings where continuous surveillance is infeasible. This approach assesses a standardized set of WHO moments during defined care episodes rather than counting all opportunities, prioritizing pragmatic process monitoring over epidemiological precision.

### 2.2. Sample and Sampling Strategy

The observation episodes constituted the unit of analysis. An episode was operationally defined as a discrete, continuous sequence of care provided to a single patient by a single healthcare worker during one patient encounter (e.g., administering intravenous medications, performing dressing changes, conducting respiratory suctioning, adjusting mechanical ventilator settings, drawing blood samples, and inserting urinary catheters). Each episode was scored across all five WHO moments to create a standardized assessment unit, regardless of which moments were applicable during a specific care interaction.

Audits were conducted weekly over eight weeks: pre-intervention (weeks 1–4) and post-intervention (weeks 5–8). Each phase included 471 episodes, yielding 2355 total moment assessments (5 moments × 471 episodes). Audits were conducted during peak clinical activity periods—08:00–12:00 (morning rounds, medication administration, routine assessments) and 14:00–17:00 (afternoon procedures and medication cycles)—when the highest volume of direct patient care procedures occurred. The audit sessions spanned all three shifts proportionally and lasted 60–90 min per session.

Auditors used convenience sampling by selecting the first available care episode during the observation window. When multiple healthcare workers were simultaneously engaged in patient care, auditors prioritized the most complex care sequences (e.g., invasive procedures, multi-step interventions) to maximize the likelihood of observing all five WHO moments and ensure representative sampling of high-intensity ICU tasks. Episode volumes and staff category distribution (nurses, interns, and nursing students) were matched across phases to minimize potential confounding by secular trends in workload or staffing patterns [22]. The two phases were analyzed as independent, cross-sectional samples.

Baseline audits (weeks 1–4) identified patterns of compliance heterogeneity across the five WHO moments. Preliminary review revealed lower adherence in awareness-dependent moments (Moment 1: before touching the patient; Moment 5: after touching patient surroundings) compared to procedurally salient, high-risk moments (e.g., Moment 3: after body fluid exposure risk), consistent with the hypothesis that compliance barriers in these moments reflected situational awareness and moment recognition deficits rather than technical skill limitations or supply availability problems [11,22]. Detailed baseline compliance rates are reported in Section 3.1.

### 2.3. Measurement Instrument and Scoring Protocol

Hand hygiene compliance was assessed using an episode-based binary checklist (compliant/non-compliant per moment) aligned with the WHO’s Five Moments of Hand Hygiene framework. This instrument minimizes subjectivity and supports standardized process monitoring in the field. Each of the five WHO moments was evaluated for every episode.

Moment applicability determination: A WHO moment was deemed “non-applicable” if the specific clinical interaction did not create a situation requiring that hand hygiene action:

- Moment 1 (Before touching the patient): Not applicable if the healthcare worker entered only to adjust equipment without direct patient contact.
- Moment 2 (Before clean/aseptic procedure): Non-applicable during routine vital sign checks or medication administration that does not involve aseptic technique.
- Moment 3 (After body fluid exposure risk): Not applicable when care involved no contact with blood, body fluids, mucous membranes, or non-intact skin.
- Moment 4 (After touching the patient): Non-applicable when the healthcare worker interacted with equipment or surroundings but never made physical contact with the patient.
- Moment 5 (After touching the patient’s surroundings): Non-applicable if the healthcare worker touched only the patient directly without contact with bed rails, monitors, tables, or other environmental surfaces.

Consistent with the institutional quality improvement practice established before this study, non-applicable moments were scored as “compliant by default” to maintain a stable, fixed denominator across all episodes. This simplified operational auditing but introduced a methodological trade-off: the composite compliance metric became a mixture of true adherence when moments were applicable and the frequency of moment applicability. This approach inflates the absolute compliance percentages compared to opportunity-based methods that exclude non-applicable moments entirely. The non-applicability rates for each WHO moment in both study phases are reported.

### 2.4. Audit Procedures and Quality Assurance

Two infection prevention staff members conducted the audits. Both auditors had ≥2 years of prior hand hygiene audit experience within the institution, applied identical procedures across the pre-intervention and post-intervention phases, and were independent of intervention planning, delivery, education sessions, and data analysis. Institutional leadership provided standardized training, emphasizing objective checklist application and contemporaneous documentation. The auditors completed paper-based checklists in real time during the observations to minimize recall bias. The leadership reviewed all completed checklists for completeness and consistency.

Formal inter-rater reliability testing was not performed, reflecting the pragmatic quality improvement constraints. Operational consistency across phases was prioritized through standardized procedures, identical auditor assignments, and supervision review, consistent with the SQUIRE 2.0 guidance on pragmatic quality improvement methodology [21]. ICU staff were aware that periodic hand hygiene audits were conducted as part of routine institutional infection control surveillance; however, specific audit dates, times, and weeks were not announced to the staff in advance to minimize acute observation reactivity. Non-blinded observation introduces the potential for observer bias and expectancy effects. Auditors were aware of the study phases and intervention implementation, which could influence the scoring, even with standardized binary checklists. The absence of formal inter-rater reliability testing further limits the confidence that the observed changes reflect true compliance shifts rather than observer drift or the evolving interpretation of checklist criteria. These methodological limitations and their implications for interpreting the magnitude of the observed changes are discussed in Section 4.5.3 and Section 4.5.7.

### 2.5. Intervention Design and Implementation

A low-cost multimodal intervention was implemented during the transition week between the pre- and post-intervention phases. The intervention targeted the two awareness-dependent moments identified as the lowest at baseline (Moments 1 and 5) and emphasized feasibility, workflow integration, and sustainability without requiring new equipment, additional staffing, or electronic monitoring systems [10,12,23].

Intervention components with quantified exposure: Targeted education huddles covered the WHO Five Moments framework content with specific emphasis on recognition of Moments 1 and 5, demonstration of proper hand hygiene technique, and interactive return demonstrations. These 15 min “safety huddles” were conducted at shift change, with four sessions per week for two consecutive weeks (eight total sessions during the transition week and first post-intervention week). All ICU nursing staff, interns, and nursing students were invited to attend at least one education session through shift-based scheduling, with a target of reaching ≥80% of eligible personnel. The actual attendance is reported in Section 3.1. Laminated World Health Organization (WHO) Five Moments pocket cards were distributed to all attendees.

The point-of-care visual reminders consisted of five large-format (24″ × 36″) full-color WHO “My Five Moments for Hand Hygiene” posters [5]. One poster was placed at the ICU unit entrance at eye level, adjacent to the hand sanitizer dispenser, and four posters were positioned at the foot of each bed cluster (three beds per cluster), ensuring visibility from the nursing stations and patient bedsides. Posters were installed at the start of the post-intervention phase and remained in place throughout weeks 5–8.

Audit feedback and reinforcement included weekly compliance summaries showing the overall compliance percentage and moment-specific breakdown with visual trend graphs, posted on the staff bulletin board (centrally located in the nurses’ break room) every Monday morning. The ICU Lead Nurse provided 5 min verbal briefings during morning shift handover (07:00–07:15) each Monday, highlighting compliance trends and reinforcing non-punitive, quality improvement messaging. Feedback emphasizes progress and collective improvement and avoids individual performance attribution or punitive framing.

Leadership engagement and role modeling included visible endorsement by the ICU Medical Director and Lead Nurse, who verbally endorsed the initiative during education huddles. Senior clinical staff demonstrated hand hygiene during clinical rounds with explicit narration of the WHO moments. Leadership authorized protected time for education sessions and ensured adequate hand hygiene supply, confirming that alcohol-based hand rub dispensers were functional and stocked throughout the study period.

No new equipment, staffing, or electronic monitoring systems were introduced. All intervention components utilized existing resources and were designed for integration into routine ICU workflows to support sustainability beyond the study’s duration.

### 2.6. Outcomes

The primary outcome was the composite hand hygiene compliance, calculated as the proportion of compliant moment assessments across all five WHO moments and episodes. The secondary outcomes were moment-specific compliance rates using fixed episode-based denominators (471 episodes per phase).

No patient-level clinical outcomes (e.g., healthcare-associated infection rates, ICU length of stay, and mortality) were measured or analyzed. The study focused exclusively on process-level adherence as an intermediate outcome, with the understanding that hand hygiene compliance is a well-established proxy for infection prevention efforts, but that short-term compliance changes cannot be directly linked to clinical outcomes without longer observation periods and larger sample sizes [24].

### 2.7. Data Analysis

Compliance was summarized as proportions with exact Clopper–Pearson 95% confidence intervals. This study employed a descriptive analytical approach consistent with quality improvement methodology and did not perform inferential statistical comparisons (e.g., hypothesis testing, *p*-values) between the pre- and post-intervention phases. The single-cycle pre-post design without concurrent controls precludes formal causal inference, and the SQUIRE 2.0 guidelines recommend descriptive reporting with confidence intervals for such quality improvement projects [21].

The analysis emphasizes the magnitude and pattern of the observed changes across WHO moments rather than statistical significance or generalizability. Confidence intervals provide precision estimates for the observed proportions but do not account for within-episode clustering (discussed in Section 4.5.7).

Sensitivity analysis. To address the risk of inflated compliance estimates from the “compliant by default” scoring rule and clarify the distinction between adherence patterns and applicability frequency, we performed a sensitivity analysis that excluded all non-applicable moments and reported “pure” adherence rates based only on the applicable opportunities. This approach assesses whether the observed changes reflect genuine behavioral shifts or artifacts of the scoring rule. The results are reported in Section 3.6.

### 2.8. Ethical Considerations

The Institutional Ethical Review Board approved the project as a quality improvement (Review No. 2269/Ethical Board/GKMC, 3 August 2023). No patient identifiers or clinical outcomes were documented. The project qualified as a quality improvement project because it (1) collected no patient data, (2) evaluated institutional processes, (3) aimed to improve existing practice rather than test hypotheses, and (4) served local improvement rather than generalizable knowledge production. Written staff consent was not required according to the institutional QI policy.

## 3. Results

### 3.1. Observation Volume and Staff Participation

The weekly audit volume and staff category distribution were matched across the study phases (Table 1). Each phase included 471 observation episodes: 214 involving registered nurses and 257 involving nursing students. Weekly episode counts ranged from 95 to 127 per week, with identical distribution patterns in the pre-intervention and post-intervention phases to minimize confounding by secular trends in workload or staffing composition.

Education sessions achieved 90% attendance among eligible ICU personnel: 27 of 30 registered nurses (90%) and 18 of 20 nursing students/interns (90%) attended at least one 15 min safety huddle during the two-week education period.

### 3.2. Baseline Compliance Patterns and Intervention Targeting Rationale

Baseline audits (Weeks 1–4) revealed heterogeneous compliance patterns across the five WHO moments (pre-intervention). Compliance was substantially higher for high-risk, procedurally salient moments: 80.0% (377/471; 95% CI 76.1–83.6) for Moment 3 (after body fluid exposure risk) and 79.0% (372/471; 95% CI 75.0–82.6) for Moments 2 and 4 (before aseptic procedures and after touching the patient, respectively).

In contrast, awareness-dependent moments requiring anticipatory recognition showed markedly lower baseline adherence: 45.0% (212/471; 95% CI 40.5–49.6) for Moment 1 (before touching the patient) and 33.0% (155/471; 95% CI 28.7–37.4) for Moment 5 (after touching the patient surroundings). This pattern is consistent with compliance barriers reflecting situational awareness and moment recognition deficits rather than technical skill limitations or supply availability problems, supporting the intervention’s targeted focus on education and environmental cues for Moments 1 and 5.

### 3.3. Frequency of Non-Applicable Moments

The non-applicability rates varied substantially across the WHO moments and remained stable between the study phases (Table 2). Moments involving aseptic procedures and body fluid exposure (Moments 2 and 3) showed the highest non-applicability rates (39–45%), whereas patient contact moments (Moments 1 and 4) were applicable in over 90% of episodes. Moment 5 (after touching the patient’s surroundings) showed intermediate non-applicability (approximately 20%). This variability directly influences composite compliance interpretation, as moments with frequent non-applicability contribute disproportionately when scored as “compliant by default.”

### 3.4. Primary Outcome: Composite Hand Hygiene Compliance

Composite compliance increased from 63.1% pre-intervention (1488/2355; 95% CI 61.2–65.0) to 82.0% post-intervention (1931/2355; 95% CI 80.4–83.5), representing an absolute increase of 18.9 percentage points (Table 3).

**Figure 1:**
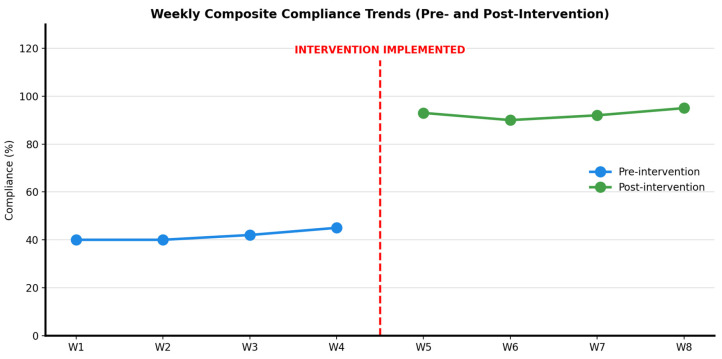
Weekly composite hand hygiene compliance during the eight-week study period. The pre-intervention (Weeks 1–4) and post-intervention (Weeks 5–8) are shown. Graphs are presented as descriptive visualizations to illustrate stability patterns within each phase and are not intended as formal interrupted time-series analyses. Error bars represent the 95% confidence interval. The auditor’s identity, procedures, and checklists remained unchanged across the study phases.

Pre-intervention compliance showed minimal week-to-week variation (range 62.1–64.5%; mean 63.1%) with no declining trend over the four-week baseline period. Post-intervention compliance remained consistently elevated across Weeks 5–8 (range 81.3–82.7%; mean 82.0%) without evidence of immediate decay. The narrow post-intervention range (1.4 percentage points) indicates stability during the brief four-week observation period (Figure 1).

### 3.5. Secondary Outcome: Moment-Specific Compliance Patterns

**Figure 2:**
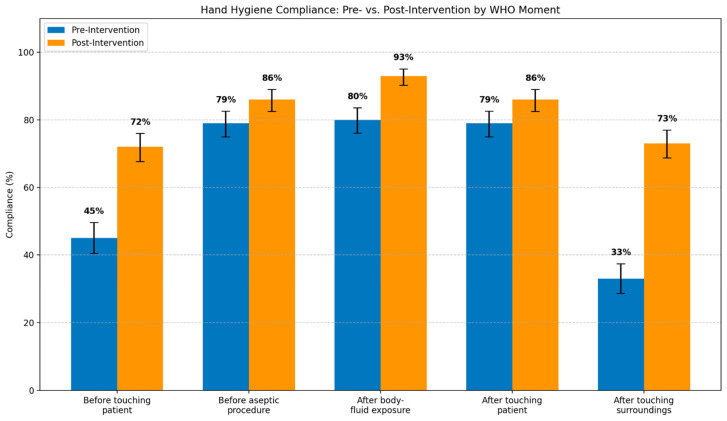
Hand hygiene compliance by WHO Five Moments before and after intervention.

Moment-specific compliance improved across all five WHO moments, with the magnitude of improvement varying by moment type (Table 4 and Figure 2). The largest absolute increases occurred in the two awareness-dependent moments explicitly targeted by the intervention: Moment 5 (after touching patient surroundings) increased by 40.0 pp, and Moment 1 (before touching patient) increased by 27.0 pp.

Moments with higher baseline compliance showed smaller absolute gains: Moment 3 (after body fluid exposure risk) +13.0 pp; Moment 2 (before aseptic procedure) +7.0 pp; and Moment 4 (after touching patient) +7.0 pp. This pattern of selective improvement, with the largest gains concentrated in intervention-targeted behaviors, is consistent with a genuine behavioral response to the education and visual cue components rather than a uniform elevation across all moments.

Figure 2: Hand hygiene compliance by WHO Five Moments before and after the multimodal intervention. Points represent compliance proportions using fixed episode-based denominators (471 episodes per phase); error bars indicate the exact 95% Clopper–Pearson confidence intervals. The values correspond to those in Table 4. These compliance percentages include non-applicable moments scored as “compliant by default” and therefore represent a mixture of true adherence and moment applicability frequency (see Table 2 for non-applicability rates and Table 5 for pure adherence excluding non-applicable moments).

### 3.6. Sensitivity Analysis: Pure Adherence Excluding Non-Applicable Moments

To address potential inflation from the “compliant by default” scoring rule, a sensitivity analysis calculated “pure adherence” by excluding all non-applicable moments and using only actual applicable opportunities as denominators (Table 5). Composite pure adherence (aggregated across all five moments and both phases) increased from 54.2% pre-intervention (801/1479 applicable opportunities) to 82.5% post-intervention (1229/1489 applicable opportunities), representing an absolute increase of 28.3 percentage points. While baseline pure adherence was 9 pp lower than the primary composite compliance metric (54.2% vs. 63.1%), post-intervention pure adherence (82.5%) converged closely with the primary compliance metric (82.0%). The direction and magnitude of improvement remained robust across both analytical approaches, indicating that the observed changes reflected genuine behavioral shifts rather than artifacts of the scoring rule alone.

The sensitivity analysis revealed markedly different adherence profiles for moments with high non-applicability rates. Moment 2 (before aseptic procedure) had pure adherence of only 34.0% at baseline (51/150 applicable opportunities) despite appearing as 79.0% compliant in the primary analysis; this improved to 56.0% post-intervention (+22.0 pp) in the secondary analysis. Moment 3 (after body fluid exposure) showed pure adherence of only 12.2% at baseline (11/90 applicable opportunities) despite appearing as 80.0% compliant in the primary analysis, demonstrating the largest absolute improvement in pure adherence across all five moments with a gain of 51.1 pp (12.2–63.3%). These discrepancies highlight how the “compliant by default” scoring rule can mask poor baseline performance in moments that are frequently non-applicable during routine ICU care.

In contrast, Moments 1, 4, and 5 showed minimal differences between primary compliance and pure adherence because these moments had low non-applicability rates (8–20%). For these frequently applicable moments, the primary compliance metric closely approximated the true opportunity-based adherence. The convergence of primary compliance and pure adherence for these moments is consistent with the interpretation that improvements reflect genuine behavioral changes in hand hygiene practice rather than changes in the frequency of moment applicability.

## 4. Discussion

### 4.1. Principal Findings

This quality improvement project evaluated a low-cost multimodal hand hygiene intervention in a resource-limited ICU setting. Composite compliance increased by 18.9 percentage points, from 63.1% pre-intervention to 82.0% post-intervention. Compliance improved across all five WHO moments, with the largest gains in awareness-dependent moments (before touching the patient: +27.0 pp; after touching the patient’s surroundings: +40.0 pp) and smaller gains in moments with higher baseline adherence, consistent with the ceiling effects.

Crucially, our sensitivity analysis demonstrated that while the “compliant by default” rule for non-applicable moments inflated absolute percentages, the composite pure adherence rates still showed a robust improvement from 54.2% to 82.5% (+28.3 pp). This reinforces that the observed improvement represents a genuine behavioral shift, rather than a measurement artifact. Moreover, the sensitivity analysis revealed that two high-risk moments, before aseptic procedures (Moment 2) and after body fluid exposure (Moment 3), had substantially lower baseline pure adherence (34.0% and 12.2%, respectively) than that suggested by the primary compliance metric (79.0% and 80.0%, respectively). This finding highlights how the “compliant by default” scoring rule can mask poor performance in moments of frequent non-applicability.

### 4.2. Week-by-Week Trends, Observation Reactivity, and Sustainability

#### 4.2.1. Baseline Stability and Observation Reactivity

Pre-intervention compliance showed minimal week-to-week variation (62.1–64.5%; mean 63.1%) with no declining trend over four weeks. The absence of early elevation followed by a decline argues against substantial observation-induced inflation of baseline compliance. Audits were conducted by institutional infection prevention staff using a routine, pre-existing protocol and were not introduced as novel research activities. The staff were accustomed to periodic audits, which were consistent with workflow-embedded observations rather than research-driven reactivity.

#### 4.2.2. Post-Intervention Stability During Brief Observation Window

Post-intervention compliance remained consistently elevated across weeks 5–8 (81.3–82.7%; mean 82.0%) without evidence of immediate decay. The narrow post-intervention range (1.4 pp) indicates stability during the brief, four-week observation window. However, this short-term stability should not be interpreted as evidence of long-term sustainability, and compliance patterns beyond one month post-intervention are unknown. The brief follow-up period is a significant study limitation that restricts conclusions regarding durable behavior change.

If gains reflected only transient novelty or heightened auditor awareness, compliance would be expected to peak immediately after the intervention and then decline. Instead, compliance plateaued during the post-intervention phase, which was consistent with an intervention-associated behavioral response. However, we acknowledge that in a single-cycle PDSA design without a concurrent control group, we cannot definitively exclude secular trends or other temporal confounders as contributing factors to observed changes.

#### 4.2.3. Moment-Specific Patterns and Targeted Response

Compliance gains were non-uniform and clustered within the intervention-targeted WHO moments. The largest improvement occurred after touching the patient’s surroundings (+40.0 percentage points), followed by before touching the patient (+27.0 percentage points). Both behaviors were identified in baseline audits as awareness-dependent and explicitly targeted through education huddles and visual cues.

This selective improvement is consistent with the pattern-matching principle of Non-Equivalent Dependent Variables (NEDV) in quasi-experimental designs, a methodological framework developed across foundational works by Campbell et al. [25,26,27]. Under this framework, a nonequivalent dependent variable is defined as “a dependent variable that is predicted not to change because of the treatment but is expected to respond to some or all of the contextually important internal validity threats in the same way as the target outcome” [26]. When validity threats, such as maturation, secular trends, or historical effects, operate, they should influence all measured outcomes similarly and concomitantly [27]. The concentration of gains within intervention-targeted behaviors while non-targeted moments showed smaller changes, therefore providing a predictive pattern that is difficult to attribute to competing explanations [26]. This coherent pattern-matching principle suggests that the more complex the pattern successfully predicted, the less likely alternative explanations are to generate the same pattern [26]. While this pattern strengthens confidence that observed changes reflect behavioral responses to intervention components rather than general trends alone, the quasi-experimental design precludes definitive causal attribution.

#### 4.2.4. Magnitude of Improvement Relative to Hawthorne Estimates

The overall improvement (+18.9 pp in composite compliance; +28.3 pp in pure adherence) exceeded the typical Hawthorne-related increases reported in the hand hygiene literature (approximately 5–15%) [13]. Moment-specific gains ranged from +7.0 to +40.0 pp in the primary analysis, with the largest effects observed in awareness-dependent behaviors. When considering baseline stability, post-intervention plateau, moment-specific clustering, and a magnitude that surpasses typical Hawthorne estimates, the findings align more closely with behavioral responses associated with the intervention rather than solely observation artifacts.

### 4.3. Relation to Existing Literature

#### 4.3.1. Compliance Pattern Alignment

The observed patterns align with prior work showing higher adherence for visibly risky or procedurally salient moments and lower adherence for anticipatory and awareness-dependent moments [5,22]. Pre-intervention compliance using the primary metric was substantially higher for high-risk moments (80.0% for Moment 3: after body fluid exposure; 79.0% for Moments 2 and 4) than for awareness-dependent moments (45.0% for Moment 1: before touching the patient; 33.0% for Moment 5: after touching surroundings).

However, the sensitivity analysis revealed that the high “compliance” for Moments 2 and 3 in the primary analysis was largely artifactual, reflecting frequent non-applicability, rather than true adherence. Pure adherence at baseline was only 34.0% for Moment 2 and 12.2% for Moment 3, far lower than the primary compliance metric suggested. This discrepancy highlights how episode-based audits with “compliant by default” scoring can mask poor adherence in moments that are often non-applicable, a methodological insight with implications for audit design and interpretation [9].

The heterogeneous baseline pattern suggests that compliance barriers reflected situational awareness and moment recognition deficits for anticipatory moments (Moments 1 and 5), while barriers for Moments 2 and 3 likely involved both awareness deficits and the infrequent occurrence of applicable opportunities during routine ICU care, consistent with previous behavioral analyses [11,22].

#### 4.3.2. Magnitude and Multimodal Effectiveness

The observed improvement is comparable to the gains reported in other multimodal interventions, particularly in resource-constrained settings [10,12,16,17,18,28]. The intervention relied on education, visual cues, feedback, and leadership engagement without electronic monitoring or automated surveillance, reinforcing the evidence that context-adapted, workflow-embedded strategies can produce measurable adherence improvements.

The sensitivity analysis demonstrated substantial gains in pure adherence for high-risk moments: Moment 3 (after body fluid exposure) improved from 12.2% to 63.3% (+51.1 pp), and Moment 2 (before aseptic procedures) improved from 34.0% to 56.0% (+22.0 pp). These findings suggest that targeting awareness-dependent moments through education and visual cues may produce collateral benefits in other moment types, although the limited number of applicable observations for these moments (90 and 150 opportunities per phase, respectively) means that these estimates have wider confidence intervals.

#### 4.3.3. Episode-Based Audits and Process Monitoring

This study adds methodological transparency to the role of episode-based audit designs. Using a standardized checklist with fixed denominators, the audit generated stable and comparable pre- and post-data suitable for process monitoring. Although such audits do not estimate true opportunity frequency, they offer operational simplicity and feasibility and can detect meaningful pattern-level changes when applied consistently [12,21]. Systematic reviews of hand hygiene compliance measurements have highlighted the heterogeneity in audit approaches and the need for transparent reporting of methodological choices [29].

The sensitivity analysis demonstrated both the utility and limitations of episode-based audits with “compliant by default” scoring. While this approach inflates absolute compliance percentages (primary metric 63.1% vs. pure adherence 54.2% at baseline), it preserves the ability to detect directionally consistent improvement (+18.9 pp vs. +28.3 pp). Future implementations should consider reporting both metrics: the episode-based composite for operational simplicity and comparability and opportunity-based pure adherence for transparency about true performance.

#### 4.3.4. Contribution to Quality Improvement Practice

This study contributes to the literature by demonstrating three key elements: (1) the feasibility and short-term effectiveness of targeting awareness-dependent WHO moments through multimodal, low-cost interventions in resource-limited ICUs; (2) the practical utility of episode-based audits as tools for directional process monitoring within rapid-cycle quality improvement, complementary to opportunity-based methods; and (3) the methodological importance of sensitivity analyses to distinguish adherence patterns from measurement artifacts when using “compliant by default” scoring rules.

#### 4.3.5. Methodological Insights from Sensitivity Analysis

The sensitivity analysis examining pure adherence after excluding non-applicable moments yielded four key insights. First, baseline pure adherence (54.2%) was nine percentage points lower than the primary composite compliance metric (63.1%), with the largest discrepancies occurring in Moments 2 and 3 owing to their high rates of non-applicability (40–45%). This demonstrates that episode-based audits with “compliant by default” scoring can substantially inflate absolute compliance estimates.

Second, despite these lower absolute adherence rates, the improvement magnitude remained robust when non-applicable moments were excluded: +28.3 percentage points in composite pure adherence (54.2% to 82.5%) compared to +18.9 percentage points in primary composite compliance (63.1% to 82.0%). This consistency across analytical approaches strengthens the confidence that the observed changes reflect genuine behavioral responses to the intervention components rather than changes in the frequency of moment applicability.

Third, the sensitivity analysis revealed that high-risk moments showed the most dramatic improvements in terms of pure adherence. Moment 3 (after body fluid exposure) demonstrated the largest absolute gain (+51.1 pp, from 12.2% to 63.3%), revealing that this moment had very poor baseline adherence, which was masked by the “compliant by default” scoring rule. This finding has important implications for intervention targeting and audit interpretations.

Fourth, all five WHO moments showed improvement in both the primary and sensitivity analyses, providing converging evidence that the observed changes reflect genuine behavioral shifts rather than measurement artifacts or changes in care patterns that would alter moment applicability frequency. This pattern-level consistency across analytical approaches is particularly important given the quasi-experimental design’s inability to rule out all alternative explanations through design alone.

### 4.4. Practical Implications

In resource-constrained ICUs, meaningful short-term improvements in hand hygiene compliance can be achieved without the need for new equipment, additional staff, or electronic surveillance systems. Prioritizing visual cues, structured feedback, and leadership endorsement integrated into routine workflows may yield substantial gains before investing in more complex monitoring technology.

Even in higher-resource settings, the findings underscore the importance of addressing cognitive and environmental determinants, specifically moment recognition and situational awareness, alongside technical training and supply availability. The large improvements in awareness-dependent moments (Moments 1 and 5) relative to smaller changes in procedurally salient moments (Moments 2 and 4) suggest that interventions targeting recognition and environmental cueing may be particularly effective for moments that depend on anticipatory behavior rather than a reactive response to visible risk.

For audit methodology, this study demonstrates that episode-based audits with “compliant by default” scoring provide operationally feasible process monitoring but should be complemented by periodic sensitivity analyses using opportunity-based calculations to ensure that compliance improvements reflect genuine adherence gains rather than changes in the frequency of moment applicability.

### 4.5. Limitations

#### 4.5.1. Single-Cycle Design and Short Follow-Up

The single-cycle PDSA design with only four weeks of post-intervention follow-up is a significant limitation that substantially restricts inferences about its long-term sustainability. This brief observation period was determined by the pragmatic constraints of rapid-cycle quality improvement rather than the optimal study design for assessing the durability of behavior change.

The observed gains reflect short-term process changes during the immediate post-intervention period and should not be interpreted as durable practice transformations. Compliance sustainability beyond the one-month measurement window is unknown. Hand hygiene behavior change typically requires sustained reinforcement, and the decay of improvement is well documented in the literature when interventions are not maintained [24]. Without extended follow-up at 3, 6, and 12 months, we cannot assess whether compliance gains persist, plateau or decline over time.

The brief follow-up period also precludes the assessment of whether the intervention components require ongoing reinforcement or whether initial education and environmental changes produce self-sustaining practice shifts. Education sessions were concentrated in a two-week period, visual cues remained static after installation, and feedback continued only through the four-week post-intervention observation period. The sustainability of improvements beyond active intervention remains an empirical question.

Therefore, this study should be interpreted as a pilot evaluation demonstrating the short-term feasibility and immediate behavioral response to a multimodal intervention rather than as evidence of sustained practice change. Future quality improvement cycles must incorporate extended surveillance periods to establish true sustainability and identify the need for booster interventions or ongoing reinforcement. The four-week post-intervention observation represents the minimum necessary to demonstrate immediate behavioral response but falls far short of the 6–12 month follow-up required to make claims about lasting behavior change.

#### 4.5.2. Direct Observation and Hawthorne Effect

Direct observation introduces potential Hawthorne effects [13,14]. Several features likely attenuated this risk, including routine institutional audits conducted by infection prevention staff, baseline stability without declining trends, post-intervention plateau rather than immediate spike-and-decline patterns, and moment-specific rather than uniform improvements. Some observation-induced elevation likely occurred; however, the magnitude of change (+18.9 pp composite, +28.3 pp pure adherence), pattern of selective improvement in targeted moments, and stability across multiple weeks align more closely with the behavioral response to intervention components than with acute observation reactivity alone.

Nevertheless, the absence of covert observation or electronic monitoring means that we cannot quantify the magnitude of the observation bias. The improvements observed may represent a combination of genuine behavior change and increased vigilance during known audit periods.

#### 4.5.3. Observer Bias

Non-blinded audits introduce an observer bias [15]. Auditors were aware of the study phases and intervention implementation, which could influence the scoring, even with standardized binary checklists. Mitigation strategies included auditor independence from intervention delivery, standardized procedures, identical checklists across phases, real-time documentation, and supervisory review. However, the absence of formal inter-rater reliability testing further limits the confidence that observed changes reflect true compliance shifts rather than observer drift or expectancy effects. This limitation reflects pragmatic quality improvement constraints but weakens the certainty of the magnitude estimates.

#### 4.5.4. Episode-Based Audit Design and Measurement Validity

Episode-based audits using fixed denominators and “compliant by default” scoring for non-applicable moments introduce measurement validity concerns [9]. This approach inflates the absolute compliance percentages and creates a metric that conflates true adherence with the frequency of moment applicability. As demonstrated in the sensitivity analysis, moments with high non-applicability (Moments 2 and 3 at 40–45%) showed substantial discrepancies between primary compliance (79–80%) and pure adherence (12–34% at baseline).

Therefore, the findings reflect pattern-level adherence within a standardized audit framework and should be interpreted as associative process monitoring data rather than causal evidence or estimates of true opportunity-based hand hygiene performance. The episode-based approach provides operational feasibility and stable denominators for pre-post comparison but does not estimate the actual opportunity frequency or true adherence rates.

#### 4.5.5. Single-Site Context

The single 12-bed mixed medical–surgical ICU in a tertiary hospital in Pakistan limits the generalizability of the findings. The applicability of these findings to other settings depends on multiple contextual factors, including baseline compliance levels, staffing patterns, workload, ICU type and patient acuity, organizational culture, existing audit practices, resource availability, and leadership engagement. The intervention’s effectiveness may differ in ICUs with different baseline adherence patterns, higher or lower acuity levels, staffing models, or varying degrees of leadership support.

#### 4.5.6. Sustainability Beyond Measurement Period

Sustainability claims are intentionally limited. Although week-to-week stability across Weeks 5–8 suggests no immediate decay during the post-intervention observation window, compliance patterns beyond one month are unknown. A longer follow-up period at 3, 6, and 12 months is required to assess the persistence of improvements, identify potential decay patterns, and determine whether booster interventions or ongoing reinforcement strategies are necessary to maintain gains.

#### 4.5.7. Statistical Dependence and Clustering

Scoring multiple WHO moments within a single care episode violates the assumption of independence of the data. This within-episode correlation or clustering effect can substantially affect precision. Even small intraclass correlation coefficients (ICC) can meaningfully inflate the design effect, meaning that observations are less independent than assumed and the effective sample size is smaller than the raw number of observations [30,31]. For example, if hand hygiene opportunities cluster within healthcare workers or care episodes, compliance behaviors may be more similar within clusters than between them, reducing the amount of truly independent information in the dataset.

Because simple binomial confidence intervals were used, the uncertainty around the point estimates may have been underestimated. While the present study prioritized descriptive process monitoring consistent with pragmatic quality improvement methodology, future work should apply generalized estimating equations (GEE) or mixed-effects models with random effects for healthcare workers and clinical units to appropriately model the correlated observations [32,33]. These approaches would provide more accurate precision estimates by accounting for within-episode clustering, repeated observations by the same auditors, and potential clustering at the healthcare worker or unit level.

#### 4.5.8. Measurement Bias from “Compliant by Default” Scoring Rule

The institutional “compliant-by-default” rule for non-applicable moments simplified operational auditing but introduced measurement bias that inflated composite compliance relative to standard WHO opportunity-based methods. The primary composite compliance metric (63.1% pre-intervention, 82.0% post-intervention) was substantially higher than pure adherence, excluding non-applicable moments (54.2% pre-intervention, 82.5% post-intervention).

More importantly, the sensitivity analysis revealed that this scoring rule masked poor baseline adherence for high-risk moments: Moment 3 (after body fluid exposure) appeared as 80.0% compliant in the primary analysis but had only 12.2% pure adherence when non-applicable episodes were excluded from the analysis. Similarly, Moment 2 (before aseptic procedures) appeared as 79.0% compliant but had only 34.0% adherence.

However, sensitivity analyses demonstrated that, although absolute adherence percentages decreased when non-applicable moments were excluded, the direction and magnitude of improvement remained consistent across both analytical approaches. This indicates that while the “compliant by default” rule inflates absolute percentages, it preserves the ability to detect meaningful behavior change patterns. This approach provides operational feasibility within episode-based audit workflows while requiring transparent reporting of sensitivity analyses to distinguish adherence patterns from the measurement artifacts.

## 5. Conclusions

Low-cost multimodal interventions embedded within routine ICU workflows can produce meaningful short-term improvements in hand hygiene compliance in resource-limited settings. Targeting awareness-dependent behaviors through education, point-of-care cues, feedback, and leadership engagement improved pure adherence from 54.2% to 82.5% over four weeks, without reliance on electronic monitoring systems. Sensitivity analyses confirmed that the observed changes reflected genuine behavioral shifts rather than measurement artifacts alone.

However, there are critical limitations to this interpretation. The brief four-week post-intervention observation period precludes conclusions about long-term sustainability, and compliance beyond one month is unknown. These findings represent process improvement measured through adherence patterns and should not be extrapolated to clinical outcomes, such as infection rates, without additional evidence. The episode-based audit design with “compliant by default” scoring provides operational feasibility but inflates absolute compliance percentages and masks true adherence for moments with frequent non-applicability. Generalizability is limited by the single-site design, resource constraints, and organizational context of this 12-bed ICU.

Within these constraints, this study provides a feasible and replicable framework for initiating hand hygiene improvement in low-resource settings, demonstrating that meaningful short-term gains can be achieved without expensive monitoring technology. The methodology contributes to the transparency of episode-based audits and the importance of sensitivity analyses to distinguish measurement artifacts from genuine adherence changes. Future work should incorporate extended follow-up periods (6–12 months minimum) to assess sustainability, link process measures to clinical outcomes, evaluate effectiveness across diverse settings, and employ sophisticated statistical methods (GEE or mixed-effects models) that account for clustering effects. Until such evidence accumulates, these findings should be interpreted as pilot data demonstrating short-term feasibility, rather than as definitive evidence supporting broad implementation or sustained behavior change.

## Figures and Tables

**Table 1:** Weekly audit volume by staff category.

Period	Week	Nurses	Nursing Students	Total Episodes
Pre	1	55	70	125
Pre	2	40	55	95
Pre	3	60	67	127
Pre	4	59	65	124
**Pre total**	**1–4**	**214**	**257**	**471**
Post	5	55	70	125
Post	6	40	55	95
Post	7	60	67	127
Post	8	59	65	124
**Post total**	**5–8**	**214**	**257**	**471**

Note: Weekly audit episode counts and staff category distribution were matched across phases to minimize potential confounding factors, such as secular trends in staffing or workload patterns.

**Table 2:** Frequency of non-applicable WHO moments by study phase.

WHO Moment	Pre-Intervention Non-Applicable n/471 (%)	Post-Intervention Non-Applicable n/471 (%)
Moment 1: Before touching the patient	38 (8.1%)	35 (7.4%)
Moment 2: Before clean/aseptic procedure	212 (45.0%)	207 (43.9%)
Moment 3: After body fluid exposure risk	188 (39.9%)	183 (38.9%)
Moment 4: After touching the patient	42 (8.9%)	40 (8.5%)
Moment 5: After touching patient surroundings	94 (20.0%)	89 (18.9%)

Note: These frequencies illustrate the variability in the moment applicability across different types of ICU care episodes. Moments 2 and 3 (aseptic procedures and body fluid exposure) were non-applicable in approximately 40–45% of observed episodes, whereas patient contact moments (1 and 4) were applicable in over 90% of episodes. Non-applicable moments were scored as “compliant by default” in the primary analysis (Section 3.4). The sensitivity analysis (Section 3.6) excluded these observations to report pure adherence rates.

**Table 3:** Composite hand hygiene compliance by study phase and week.

Phase	Week	Episodes (n)	Moment Assessments (n)	Compliant (n)	Compliance (%)	95% CI
Pre-Intervention						
Pre	1	125	625	388	62.1	58.1–66.0
Pre	2	95	475	295	62.1	57.6–66.4
Pre	3	127	635	403	63.5	59.7–67.1
Pre	4	124	620	402	64.5	60.6–68.2
Pre total	1–4	471	2355	1488	63.1	61.2–65.0
Post-Intervention						
Post	5	125	625	512	81.9	78.5–85.0
Post	6	95	475	386	81.3	77.4–84.7
Post	7	127	635	520	81.9	78.5–85.0
Post	8	124	620	513	82.7	79.4–85.7
Post total	5–8	471	2355	1931	82.0	80.4–83.5

Note: The denominator for overall compliance = 5 WHO moments × 471 episodes = 2355 total moment assessments per phase. Compliance percentages reflect the proportion of compliant assessments out of total assessments, including non-applicable moments scored as “compliant by default” per institutional protocol. Confidence intervals were exact Clopper–Pearson binomial intervals.

**Table 4:** Hand hygiene compliance by WHO Five Moments.

WHO Moment	Pre-Intervention	95% CI	Post-Intervention	95% CI	Absolute Change (pp)
Moment 1: Before touching patient	212/471(45.0%)	40.5–49.6	339/471(72.0%)	67.7–76.0	+27.0
Moment 2: Before aseptic procedure	372/471(79.0%)	75.0–82.6	405/471 (86.0%)	82.5–89.0	+7.0
Moment 3: After body fluid exposure	377/471 (80.0%)	76.1–83.6	438/471 (93.0%)	90.3–95.1	+13.0
Moment 4: After touching patient	372/471(79.0%)	75.0–82.6	405/471 (86.0%)	82.5–89.0	+7.0
Moment 5: After touching surroundings	155/471(33.0%)	28.7–37.4	344/471 (73.0%)	68.8–77.0	+40.0

Note: Moment-specific compliance rates use a fixed episode-based denominator (471 episodes per phase) and include non-applicable moments scored as “compliant by default.” These percentages are not true opportunity-based adherence rates and cannot be interpreted as the proportion of applicable moments in which hand hygiene was performed. Compliance rates for Moments 2 and 3 are particularly inflated because these moments were non-applicable in 40–45% of episodes (see Table 2). For example, the 79.0% pre-intervention “compliance” for Moment 2 reflects a mixture of 51 truly compliant observations among 150 applicable opportunities plus 321 episodes scored as compliant by default (see sensitivity analysis, Table 5, for pure adherence rates). The moment-level results are not additive and should not be summed.

**Table 5:** Sensitivity analysis: Pure adherence excluding non-applicable moments.

WHO Moment	Phase	Total Episodes (N)	Non-Applicable (n)	Actual Opportunities (n)	Compliant (n)	Pure Adherence (%)	95% CI
Moment 1:Before touching patient	Pre	471	38	433	212	49.0%	44.2–53.8
	Post	471	35	436	339	77.8%	73.7–81.5
Moment 2:Before aseptic procedure	Pre	471	321	150	51	34.0%	26.5–42.2
	Post	471	321	150	84	56.0%	47.7–64.1
Moment 3:After body fluid exposure	Pre	471	381	90	11	12.2%	6.3–20.8
	Post	471	381	90	57	63.3%	52.5–73.2
Moment 4:After touching patient	Pre	471	42	429	372	86.7%	83.1–89.8
	Post	471	40	431	405	94.0%	91.3–96.0
Moment 5:After touching surroundings	Pre	471	94	377	155	41.1%	36.2–46.2
	Post	471	89	382	344	90.1%	86.6–92.9
COMPOSITE (all moments)	Pre	2355	876	1479	801	54.2%	51.6–56.7
	Post	2355	866	1489	1229	82.5%	80.5–84.4

Note: Pure adherence represents the proportion of applicable hand hygiene opportunities where compliance was observed, excluding all non-applicable moments. For Moments 1, 4, and 5, non-applicability was infrequent (<20%); therefore, pure adherence closely approximated the primary compliance metric. For Moments 2 and 3, which were non-applicable in 40–45% of episodes, pure adherence revealed substantially lower baseline performance (34.0% and 12.2%, respectively) than that suggested by the primary analysis (79.0% and 80.0%, respectively). The composite pure adherence aggregates across all time periods and both phases.

## Data Availability

The data presented in this study are available from the corresponding author upon reasonable requests. The data are not publicly available due to institutional quality improvement policies and staff confidentiality.

## Supplementary Materials

The following supporting information can be downloaded at: https://www.mdpi.com/article/10.3390/healthcare14030363/s1, SQUIRE 2.0 Reporting Checklist.

## Abbreviation

CI	Confidence Interval
CLABSI	Central line-associated bloodstream infection
ICC	Intraclass Correlation Coefficient
ICU	Intensive Care Unit
NEDV	Non-Equivalent Dependent Variables
PDSA	Plan–Do–Study–Act
pp	percentage points
QI	Quality Improvement
SQUIRE	Standards for QUality Improvement Reporting Excellence
VAP	Ventilator-Associated Pneumonia
WHO	World Health Organization

## References

[1] Zaidi A.K.M., Huskins W.C., Thaver D., Bhutta Z.A., Abbas Z., Goldmann D.A. (2005). Hospital-acquired neonatal infections in developing countries. Lancet.

[2] Mathur P. (2011). Hand hygiene: Back to the basics of infection control. Indian J. Med. Res..

[3] Boyce J.M., Pittet D. (2002). Guideline for hand hygiene in health-care settings. MMWR Recomm. Rep..

[4] World Health Organization (2009). WHO Guidelines on Hand Hygiene in Health Care: First Global Patient Safety Challenge—Clean Care Is Safer Care.

[5] Sax H., Allegranzi B., Uçkay I., Larson E., Boyce J., Pittet D. (2007). My five moments for hand hygiene. J. Hosp. Infect..

[6] Umscheid C.A., Mitchell M.D., Doshi J.A., Agarwal R., Williams K., Brennan P.J. (2011). Estimating preventable healthcare-associated infections. Infect. Control Hosp. Epidemiol..

[7] Abbas S. (2024). Challenges of implementing infection prevention and antimicrobial stewardship programs in resource-constrained settings. Antimicrob. Steward. Healthc. Epidemiol..

[8] Tschudin-Sutter S., Pargger H., Widmer A.F. (2010). Hand hygiene in the intensive care unit. Crit. Care Med..

[9] Livorsi D.J., Goedken C.C., Sauder M., Vander Weg M.W., Perencevich E.N., Reisinger H.S. (2018). Barriers to audit-and-feedback programs using direct observation of hand hygiene. JAMA Netw Open..

[10] Pittet D., Hugonnet S., Harbarth S., Mourouga P., Sauvan V., Touveneau S., Perneger T.V. (2000). Effectiveness of a hospital-wide programme to improve hand hygiene. Lancet.

[11] Michie S., van Stralen M.M., West R. (2011). The behaviour change wheel. Implement. Sci..

[12] Ivers N., Yogasingam S., Lacroix M., Brown K.A., Antony J., Soobiah C., Simeoni M., Willis T.A., Crawshaw J., Antonopoulou V. (2025). Audit and feedback: Effects on professional practice. Cochrane Database Syst. Rev..

[13] Hagel S., Reischke J., Kesselmeier M., Winning J., Gastmeier P., Brunkhorst F.M., Scherag A., Pletz M.W. (2015). Quantifying the Hawthorne effect in hand hygiene compliance. Infect. Control Hosp. Epidemiol..

[14] Bruchez S.A., Duarte G.C., Sadowski R.A., da Silva Filho A.C., Fahning W.E., Nishiyama S.A., Tognim M.C., Cardoso C.L. (2020). Assessing the Hawthorne effect on hand hygiene compliance in an ICU. Infect. Prev. Pract..

[15] Dhar S., Tansek R., Toftey E.A., Dziekan B.A., Chevalier T.C., Bohlinger C.G., Fitch M., Flanagan M.E., Chopra T., Marchaim D. (2010). Observer bias in hand hygiene compliance reporting. Infect. Control Hosp. Epidemiol..

[16] Mouajou V., Adams K., DeLisle G., Quach C. (2022). Hand hygiene compliance and prevention of hospital-acquired infections. J. Hosp. Infect..

[17] Sandbekken I.H., Utne I., Hermansen Å., Grov E.K., Løyland B. (2024). Multimodal interventions and hand hygiene adherence in nursing homes. Am. J. Infect. Control..

[18] Chaudhary P., Gupta V. (2024). Hand hygiene interventions in a tertiary care institute. J. Prev. Med. Hyg..

[19] Elbahr U., Alwazzan N., Reyes C.S., Pastrana J., Vineeth C., Sipahi H., Sipahi O.R. (2025). Hand hygiene compliance in a tertiary-care hemato-oncology hospital. Mediterr. J. Infect. Microbes. Antimicrob..

[20] Eco S., Petcka N.L., Li K., Hechenbleikner E.M. (2025). Quality improvement in rural and low-resource settings. Am. Surg..

[21] Ogrinc G., Davies L., Goodman D., Batalden P., Davidoff F., Stevens D. (2016). SQUIRE 2.0 guidelines. BMJ Qual. Saf..

[22] Afework A., Tamene A. (2025). Barriers to hand hygiene adherence. BMC Infect. Dis..

[23] Loftus M.J., Guitart C., Tartari E., Stewardson A.J., Amer F., Bellissimo-Rodrigues F., Lee A.F., Mehtar S., Sithole B.L., Pittet D. (2019). Hand hygiene in low- and middle-income countries. Int. J. Infect. Dis..

[24] Lotfinejad N., Peters A., Tartari E., Fankhauser-Rodriguez C., Pires D., Pittet D. (2021). Hand hygiene in health care: 20 years of ongoing advances and perspectives. Lancet Infect. Dis..

[25] Campbell D.T., Stanley J.C. (1966). Experimental and Quasi-Experimental Designs for Research.

[26] Shadish W.R., Cook T.D., Campbell D.T. (2002). Experimental and Quasi-Experimental Designs for Generalized Causal Inference.

[27] Coryn C.L.S., Hobson K.A. (2011). Using nonequivalent dependent variables to reduce internal validity threats in quasi-experiments: Rationale, history, and examples from practice. New Dir. Eval..

[28] Allegranzi B., Gayet-Ageron A., Damani N., Bengaly L., McLaws M.L., Moro M.L., Memish Z., Urroz O., Richet H., Storr J. (2013). Global implementation of WHO’s multimodal strategy for improvement of hand hygiene: A quasi-experimental study. Lancet Infect. Dis..

[29] Gould D.J., Moralejo D., Drey N., Chudleigh J.H., Taljaard M. (2017). Interventions to improve hand hygiene compliance in patient care. Cochrane Database Syst. Rev..

[30] Kerry S.M., Bland J.M. (1998). Statistics notes: The intracluster correlation coefficient in cluster randomisation. BMJ.

[31] Fournel I., Tiv M., Hua C., Soulias M., Astruc K., Aho L.S. (2010). Randomisation and sample size for clinical audit on infection control. J. Hosp. Infect..

[32] Huis A., Schoonhoven L., Grol R., Donders R., Hulscher M., van Achterberg T. (2013). Impact of a team and leaders-directed strategy to improve nurses’ adherence to hand hygiene guidelines: A cluster randomised trial. Int. J. Nurs. Stud..

[33] Sickbert-Bennett E.E., DiBiase L.M., Schade Willis T.M., Wolak E.S., Weber D.J., Rutala W.A. (2016). Reduction of healthcare-associated infections by exceeding high compliance with hand hygiene practices. Emerg. Infect. Dis..

