# Supplementary material for: Improving Hand Hygiene Compliance in a Resource-Limited ICU Using a Low-Cost Multimodal Quality Improvement Intervention
Source: Healthcare (Basel). 2026 Jan 30;14(3):363. doi: 10.3390/healthcare14030363 (PMC12897775; doi:10.3390/healthcare14030363)
Supplement: Supplementary file 1 [file healthcare-14-00363-s001.zip › healthcare-4100307-supplementary.pdf]

SQUIRE 2.0 Reporting Checklist

Manuscript title: Improving Hand Hygiene Compliance in a Resource-Limited ICU Using a Low-Cost Multimodal Quality Improvement Intervention

Title & Abstract

SQUIRE Item	Description	Location in Manuscript
1	Title identifies study as quality improvement	Title page
2a	Abstract summary	Abstract
2b	Problem, intervention, methods, results	Abstract
2c	Key outcomes	Abstract (Highlights section)

Introduction

SQUIRE Item	Description	Location
3	Problem description	Introduction, Section 1, paragraphs 1-3
4	Available knowledge	Introduction, Section 1, paragraphs 1-3
5	Rationale (behavioral + audit logic)	Section 1.1 (Study Rationale and Contributions)
6	Specific aims	Section 1.2 (Study Objectives and Scope)

Methods

SQUIRE Item	Description	Location
7a	Context (ICU setting, staffing)	Section 2.1 (Study Design and Setting)
7b	Ethical considerations	Section 2.8 (Ethical Considerations)
8	Intervention description	Section 2.5 (Intervention Design and Implementation)
9a	Study of the intervention	Sections 2.1 (Study Design), 2.2 (Sample and Sampling Strategy)
9b	Measures & definitions	Sections 2.3 (Measurement Instrument), 2.6 (Outcomes)
9c	Data completeness & reliability	Section 2.4 (Audit Procedures and Quality Assurance)
10	Analysis methods	Section 2.7 (Data Analysis)

Results

SQUIRE Item	Description	Location

11a	Results of intervention	Sections 3.4 (Primary Outcome), 3.5 (Secondary Outcomes), 3.6 (Sensitivity Analysis)
11b	Process measures	Sections 3.1-3.6; Tables 1-5; Figures 1-2
11c	Contextual changes	Section 3.1 (Observation Volume and Staff Participation)

Discussion

SQUIRE Item	Description	Location
12	Summary of findings	Section 4.1 (Principal Findings)
13	Interpretation	Sections 4.2 (Week-by-Week Trends), 4.3 (Relation to Existing Literature), 4.4 (Practical Implications)
14	Limitations	Section 4.5 (Limitations, subsections 4.5.1-4.5.8)
15	Sustainability	Sections 4.2.2, 4.5.1, 4.5.6
16	Conclusions	Section 5 (Conclusions)

Other Information

SQUIRE Item	Description	Location
17	Funding	Funding statement (after Conclusions)
18	Conflicts of interest	Conflicts of Interest statement
19	Ethical approval statement	Section 2.8 (Ethical Considerations) and Institutional Review Board Statement
